# Association of Age at Menarche with General and Abdominal Obesity in Young Women

**DOI:** 10.3390/medicina60101711

**Published:** 2024-10-18

**Authors:** Rada Rakic, Tatjana Pavlica, Jelena Havrljenko, Jelena Bjelanovic

**Affiliations:** 1 Department of Biology and Ecology, Faculty of Sciences, University of Novi Sad, Trg Dositeja Obradovica 2, 21000 Novi Sad, Serbia; rada.rakic@dbe.uns.ac.rs (R.R.); jelenav@dbe.uns.ac.rs (J.H.); 2 Institute for Public Health of Vojvodina, Faculty of Medicine, University of Novi Sad, Hajduk Veljkova 3, 21000 Novi Sad, Serbia; jelena.bjelanovic@izjzv.org.rs

**Keywords:** age at menarche, general obesity, abdominal obesity, young women

## Abstract

*Background and Objectives*: Age at menarche is related to various biological and socioeconomic factors in childhood. The aim of the study was to examine the association of age at menarche with general and abdominal obesity in young women. *Materials and Methods*: A transversal anthropometric survey was conducted with 102 females from 21 to 25 years of age. The surveyed traits included height, weight, waist circumference (WC) and hip circumference (HC). General obesity was assessed using the body mass index (BMI) and abdominal obesity by WC, waist-to-hip ratio (WHR) and waist-to-height ratio (WHtR). A retrospective method was used for collecting age at menarche data. *Results*: The average age at menarche is 12.80 years. Early menarcheal age (<12 years) is detected in 25.5% of young females, while late onset of menarche (>14 years) is recorded for 20.6% of subjects. Early menarche age subjects exhibit significantly higher BMI, WC and WHtR in comparison with their late menarche age peers. There is a significant negative correlation between BMI, WC and WHtR values and menarcheal age. Late age at menarche is associated with higher probability of underweight status (BMI < 18.5 and/or WHtR < 0.4). *Conclusions*: Age at menarche has a negative correlation with general and abdominal obesity. Young women with early age at menarche show statistically higher values of BMI, WC and WHtR, while those with late menarcheal age show greater susceptibility to becoming underweight.

## 1. Introduction

Menarche is one of the major events in the reproductive part of a woman’s life. Age at menarche is taken as an indicator of life quality of a population as, among other factors, it is influenced by living conditions, nutrition and socioeconomic status. Although menarche usually occurs between the ages of 10 and 15, age at menarche is variable [1]. Due to recorded discrepancies among populations, there are no standard definitions of early and late menarche in the relevant literature. Nevertheless, early menarche is usually considered to be prior to the age of 12 [2], although some researchers define it as menarche appearing before the age of 11 [3]. The normal period of menarche occurrence is usually defined between the ages of 11 and 15 [4] or as the period between 12 and 14 years of age [5].

In recent years, menarche has become an important issue due to its possible association with health condition in adulthood [6], particularly in cases when it occurs at an early age [7]. Globally speaking, menarche today appears earlier than in previous decades [8]. Lower age at menarche has been reported in Europe [9], and a similar trend of a 3.6-month decline has been recorded in the region of Vojvodina (the Republic of Serbia) in the first two decades of the 21st century [10].

Mishra and associates state that a woman’s health condition is directly associated with the age at menarche [11]. Early menarche is known to be associated with an increased risk of chronic diseases, such as cardiovascular diseases [12], rheumatoid arthritis [13] and cancer, particularly breast cancer [14]. According to Key et al., women who experience menarche before 12 years of age have a 23% higher risk of developing breast cancer than those who first menstruate at 15 years of age or later [15]. Also, Won et al. indicate that women who experience menarche before 12 years of age show a higher incidence of metabolic syndrome and cardiovascular mortality rate [16].

Numerous longitudinal and cross-sectional studies have pointed to a potential relationship between age at menarche and high body mass index (BMI) [17] and obesity and suggest that increased adiposity in girls is associated with earlier onset of puberty and age of menarche [18,19,20,21]. Early age at menarche thus appears as a risk factor for obesity [2,22,23]. However, it is still unclear if early menarche leads to obesity or obesity causes early menarche occurrence. Some evidence shows that the association between age at menarche and body composition in adulthood is strongly related to body composition in late childhood and therefore age at menarche would be a proxy of pre-pubertal body composition [24].

A Mendelian study of randomization claims that early menarche triggers changes in body composition by means of hormone activity and psychological factors [25]. The age at menarche is associated with the level and length of estrogen exposure. Women with early onset of menarche are exposed to larger cumulative doses of estrogen and progesterone in their adulthood than those with later-onset menarche [26]. Higher concentrations of androgens that are related to early menarche occurrence can also lead to obesity [25,27]. Some longitudinal studies have shown that women who experienced early menarche had a relatively high BMI after reaching adulthood [28].

As already stated, a large number of studies have focused on the association of menarche with obesity [28,29,30]. However, the relationship between age at menarche and underweight condition also needs to be considered [5,31]. Since both the excess and lack of fat can affect health, both of these conditions need to be taken into consideration.

In light of the current downward trend in age at menarche, the relationship between early and late onset of menarche and obesity in young women seems crucial for the control of obesity in older age. To the best of our knowledge, there have been no reports on the relationship of age at menarche with general and abdominal obesity in women in the Republic of Serbia. With this in mind, the present study aims at examining the association of age at menarche and general and abdominal obesity in young women.

## 2. Materials and Methods

### 2.1. Study Design

A transversal anthropometric survey was conducted at the Department of Biology and Ecology, Faculty of Sciences, University of Novi Sad, in 2023. In total, 102 female students born between 1998 and 2002 were surveyed. The criteria for inclusion applied to third year female undergraduates attending a human biology course as part of their bachelor’s degree course in biology. The course curriculum covers most of the topics that the current paper deals with. They were between 20.50 and 25.49 years old at the time of the research and gave their consent to participate in the research. Exclusion criteria were pregnancy and lactation. The participants anonymously filled out a questionnaire (Supplementary Materials) that included information regarding date of birth, the date of first menstruation and the date of the survey.

For each subject, decimal age was calculated, based on the date of the survey and date of birth. The surveyed traits included height, weight, waist circumference (WC) and hip circumference (HC). All anthropometric traits were measured by a trained professional.

Height was surveyed by means of an anthropometer (±1 mm; SieberHegnerMaschinen AG Zürich Switzerland), with head position in the Frankfurt plane. Weight was assessed by means of a portable digital weighing scale with a level of accuracy of ±0.1 kg. Waist circumference was obtained midway between the bottom of the costal arch (*arcus costalis*) and the top of the iliac crest (*crista iliaca*). The measurements were taken in the upright standing position with slightly abducted arms, while breathing calmly, and the recorded measures were taken at the end of a normal expiration. Hip circumference was measured at the most bulging point of the greater trochanter (*trochanter major femoris*). The measurements were taken in the upright position, with arms at the sides, feet positioned close together and with relaxed gluteal muscles.

Height and weight measurements were used for assessing BMI that serves as an indicator of general obesity. The applied categories were based on the threshold values of BMI for adults [32], where subjects with BMI < 18.50 kg/m^2^ were classified as underweight, those with BMI range 18.50–24.99  kg/m^2^ as normal weight, those with BMI ranging from 25–29.99 kg/m^2^ as overweight, while those with BMI ≥ 30 kg/m^2^ were in the obese category. Abdominal obesity was assessed on the basis of WC > 80 cm, WHR > 0.8 [32] and WHtR ≥ 0.5. Values of WHtR ˂ 0.4 were classified as too low and those within the 0.40–0.49 range as normal [33].

The retrospective method was applied for assessing menarcheal age. The subjects were asked about the date of their first menstruation. The age at menarche was assessed by subtracting the date of birth from the date of menarche onset. In cases when subjects were not sure about the exact date of menarche and reported only the month and the year, the 15th day of the month was taken for calculation. If only the year of menarche was reported, the age at menarche was obtained by dividing the difference in the birth date and July 1st of the reported year by 365.25. The age at menarche categorization included early age (˂12 years), average (12–14 years) and late age (>14  years), complying with the vast number of literature reports [5,34].

### 2.2. Statistical Analysis

The results are presented as means± standard deviation (SD) or median (standard error) for numerical variables and as absolute frequencies with the set percentage values for the category variables. The effect of age at menarche categories on BMI, WC, WHR and WHtR was assessed by linear regression analysis. Logistic regression was applied for assessing the probability of obesity in relation to the age at menarche. A Mann–Whitney U test was used for determining the significance of differences between two groups.

Data processing was performed using IBM Statistics SPSS, version 23.0. Statistically significant difference was set as *p* < 0.05.

## 3. Results

The average age of subjects is 22.56 ± 0.81 years. The assessed anthropometric traits and indicators of general and abdominal obesity are within the range of normal values (Table 1).

Table 2 indicates that young women with early age at menarche exhibit smaller height but higher BMI and greater indicators of abdominal obesity (WC, WHR and WHtR), while those with later menarcheal age show smaller values of these indicators. On average, the height value is 1.1 cm lower in early age menarche females and 2.9 cm higher in those with late age menarche onset. The BMI values in early menarcheal age subjects are 2.63 kg/m^2^ higher than in other subjects. Opposite to them, females with late age at menarche have 1.86 kg/m^2^ lower BMI values in comparison with other subjects. As for waist circumference, the values are 6.23 cm higher in subjects with early menarche and 6.11 cm smaller in females with later menarcheal age. Early menarche age subjects exhibit significantly higher BMI (Z = −2.587; *p* = 0.010), WC (Z = −2.785; *p* = 0.005) and WHtR (Z = −2.111; *p* = 0.035) and smaller height (Z = −1.997; *p* = 0.045) in comparison with their late menarche peers. The WHR values are also higher in females with early age at menarche, but the difference is not statistically significant.

The distribution of general and abdominal obesity prevalence within early and late age at menarche groups (Table 3) shows that more than a fifth of the sample (22.55%) shows values of BMI ≥ 25 kg/m^2^. As for early age at menarche subjects, there is a significantly higher prevalence of BMI ≥ 25 kg/m^2^ in comparison with those who show BMI ˂ 25 kg/m^2^ values (52.17% as opposed to 17.72%, *p* = 0.000, respectively). In the second group of respondents, only two women (9.09%) have a BMI ≥ 25 kg/m^2^, and five respondents with late menarche were classified as undernourished (22.73%). The prevalence of subjects of normal weight is 68.63%, 17.65% of them are overweight, 4.90% are obese and 8.82% are underweight. Considering overweight females, 38.89% of these subjects had menarche at an early age. Early menarcheal age is also reported by all subjects within the obese group. As for underweight subjects, one of them (11.11%) reported early age at menarche, and five of them (55.56%) late menarcheal age. Considering abdominal obesity, normal weight status based on WC and WHR values equals 77.45% and 78.43%, respectively. Based on the values WC ˃ 80 cm, WHR ˃ 0.80 and WHtR ≥ 0.50, the percentage of obese subjects is 22.54%, 21.57% and 18.63%, respectively. Within this category, early age at menarche is present in 56.52%, 40.91% and 47.37%, respectively. The percentage of subjects with values WHtR < 0.4 equals 13.73%, out of which 21.43% had early menarche and 50% late menarche. The average age at menarche is 12.80 ± 1.53 years, and the median equals 12.62 years (0.15). Early menarche onset is present in 25.5% of subjects, while late age at menarche is recorded in 20.6% of females.

There are significant differences between the average age at menarche and the categories of WC (Z = −2.891; *p* = 0.004), BMI ˂ 25 and BMI ≥ 25 kg/m^2^ (Z = −3.087; *p* ˂ 0.002) and WHtR ˂ 0.5 and WHtR ≥ 0.50 (Z = −2.536; *p* = 0.011).

Linear regression analysis points to a significant inverse relationship between the age at menarche and BMI, WC and WHtR values. Subjects with early age at menarche exhibit significantly higher values of general and abdominal obesity, except for WHR values, where the relationship is not statistically significant. Height is directly related to age at menarche, but the relationship is not statistically significant (Table 4).

As observed in Table 5 where logistic regression results are presented, there is a significant negative relationship of age at menarche with BMI, WC and WHtR, i.e., there is a significantly higher probability of exhibiting obesity (BMI ≥ 25) and abdominal obesity (WC > 80 cm and WHtR ≥ 0.50) for early menarcheal age women than in other subjects. A negative association is observed in the value WHR > 0.80, but the level of probability is insignificant. In relation to later age at menarche, the BMI and WC values show an increase of 0.70 kg/m^2^ and 0.66 cm, respectively.

## 4. Discussion

The results of the present study point to a significant inverse relationship between the age at menarche and BMI, WC and WHtR in young women. The findings also point to a higher probability of general and abdominal obesity (BMI ≥ 25, WC > 80 and WHtR ≥ 0.5, respectively) among women with early menarcheal age. Contrary to this, in subjects with later age at menarche the probability of underweight status is greater (BMI < 18.5; WHtR < 0.4).

The current study reports the average menarcheal age of 12.80 years, which is lower than the reported value of 13.1 years in Saudi Arabia [30] and 15.4 years in China [35]. However, the value is greater in comparison with the average menarcheal age reported in other countries such as Italy (12.4 years [36] and 10.07 years [37]), the United Kingdom (12.5 years), [38] Kuwait (12.41 years) [39] and Australia (12.7 years) [40].

Early age at menarche prevalence is 25.5%, which is in line with the results obtained in Croatia (25%) [41], but lower in comparison with other reported studies, for example, in Brazil (35.2%) [2] and Saudi Arabia (35.6%) [30]. However, in comparison with China where the reported early age at menarche prevalence is 5.4% [35] the obtained results are considerably higher. Further, in the current study the percentage of normal age at menarche (12–14 years of age) is 52.9%, while late menarcheal age appears in 21.6% of cases, the latter value being much higher than the one recorded in Saudi Arabia where the late age at menarche prevalence equals 6% [30].

Height is directly associated with age at menarche and in this research the height value is 3 cm lower in early menarcheal age females than in their peers with late age menarche onset (166.16 as opposed to 169.21 cm). A direct association of height and age at menarche has been reported in previous studies as well [42]. Pai et al. report that early timing of puberty directly affects adult height [43]. A possible explanation is that menarche does not occur until peak height velocity has been reached [44,45]. This is the period within puberty when the growth spurt peaks and begins to decelerate. The pubertal growth spurt starts at the beginning of puberty and is caused by an increase in sex hormones, such as estrogens and androgens [46]. The cessation of growth of long bones occurs at the end of the decline in the pubertal growth spurt and is caused partly by low concentrations of estrogen [47]. Girls who reach puberty early lose pre-pubertal growth and hit peak height velocity at a younger age, which sets in motion an early stoppage of long bone growth. Girls who are late to mature experience extra pre-pubertal growth and a delayed age of peak height velocity, leading to an extended period of long bone growth.

Within the group of subjects with early menarche onset, there is a significantly higher percentage of females with values of BMI ≥ 25 kg/m^2^ than those with BMI ˂ 25 kg/m^2^ (52.17% as opposed to 17.72%, *p* = 0.000). Out of 22.55% of subjects with BMI ≥ 25 kg/m^2^ values, 52.17% are characterized with early menarcheal age and this trend is in line with recently reported findings where out of 21.7% females with BMI ≥ 25 kg/m^2^ values, 45.6% had early menarche [30]. Each one-year increase in menarcheal age in this study is associated with a decrease in BMI value by 0.70 kg/m^2^ and waist circumference by 0.66 cm. This points to an increase in BMI and waist circumference values in early age at menarche. Sumi et al. report that each one-year drop in age at menarche is associated with an increase in BMI and waist circumference by 0.25 kg/m^2^ and 0.6 cm, respectively [48]. Other studies on young females aged 17–20 years [40] point to a decrease in BMI by 0.75 kg/m^2^ in each successive menarcheal age reported. In adults, the ratio between the presence and absence of obesity is 0.82 [29] and 0.89 [49].

A previous comprehensive meta-analysis also points to a greater association of age at menarche with BMI values of young women [28]. This also supports the hypothesis that early menarche can lead to obesity in young adult women [3]. A large number of studies have reported a significant relationship between early age at menarche and greater obesity in later years in life [23,28,29,30]. However, Pierce and Leon’s study [49] points to a different role of BMI in childhood. There are also other reports on an insignificant relationship between BMI and age at menarche [50]. In our study, the age at menarche is inversely related to the BMI values in the early adulthood period, which corroborates previous research [5,23,30,51,52]. In an earlier meta-analysis the average difference in BMI in young women with early menarche onset (<12 years) in relation to the ≥12 years group was 0.34 kg/m^2^ [28], and in a recently published study [51] it equals 1.13 kg/m^2^. Yang and associates have recorded a difference of 0.7 kg/m^2^ in BMI and 1.5 cm in WC [35]. These values are lower than those obtained in the present study, with differences in BMI of 2.63 kg/m^2^ (24.83 as opposed to 22.20 kg/m^2^) and 6.23 cm regarding WC (79.38 as opposed to 73.15 cm).

A question that is raised regarding the current topic is whether greater weight predisposes earlier puberty in girls, or early puberty with its physical and hormone changes contributes to weight (fat) increase in older age [53]. Some findings suggest that overeating and obesity in early childhood can lead to early age at menarche [52,54]. There is also evidence to support the effects of higher childhood BMI on the onset and development of puberty in both boys and girls [21]. As reported by Perry et al. a genome-wide association study (GWAS) in humans identified multiple BMI-increasing alleles that are also associated with earlier age of menarche, further confirming genetic coregulation of childhood obesity and early menarche [55].

On the other hand, overweight condition in childhood and early menarcheal age increase the chances of becoming obese in young adults [56], which is indicated by the results of this investigation. Early menarche in women causes a higher incidence of obesity owing to the continuous fat accumulation induced by prolonged exposure to estrogen and adrenal steroids [57]. Nevertheless, McTique and associates claim that later age obesity is not an unusual phenomenon [22]. Some authors state that the inverse relationship of age at menarche with BMI and abdominal obesity in middle age cannot be associated with BMI values in early childhood [49]. Still, studies that predict obesity in adulthood based on childhood health status are of a limited scope and scarce.

A literature review suggests that females with late age at menarche exhibit significantly lower BMI in comparison with their peers characterized by early onset of menarche [28,30,31]. The present study also indicates that the majority of underweight subjects have menarche at a late age, and none of them reported early menarcheal age. Our findings also suggest that the probability of general and abdominal obesity is lower in women with late age at menarche. This is congruent with recent findings of Żurawiecka and Wronka [5]. Underweight condition is also concerning, as it can be related to a number of health problems, such as osteoporosis, menstrual disorders, reproductive problems and a weakened immune system [58,59]. Both early and later age at menarche females have a higher risk of developing diseases related to abnormal (insufficient or excessive) fat [28].

Although a number of studies have focused on the association of early menarche with BMI values in adult age, only a few of them have included indicators of abdominal obesity such as WC and WHR and WHtR indexes [60,61,62]. The present results point to a significant inverse correlation between the age at menarche and the current BMI, WC and WHtR values. This is in line with recent research [2] that reports on a significant inverse correlation between the age at menarche and the values of BMI and WC. Earlier research has shown that the measures reflecting abdominal obesity are significantly more common in women with early menarcheal age [63] and that early age at menarche is related to visceral subcutaneous deposits of abdominal fat in adult age [64]. The current findings support previously published results on the inverse relationship of early age at menarche with waist circumference [5,65]. The study shows no significant correlation between WHR and menarcheal age, which is in line with previous literature reports [5,50]. The results of this study suggest that females with early menarche could potentially be at increased risk for obesity in adulthood and thus represent a more vulnerable group in the population. However, considering that obesity in adulthood can be caused by other factors, and not only early menarche, one should be very careful in the application of possible exogenous interventions aimed at preventing early menarche. Therefore, the promotion of healthy lifestyle, proper nutrition and regular physical activity should be aimed at the entire population, and particularly at this group of females. Timely detection, monitoring and prevention could be the key to this potential problem. Therefore, future studies should be focused on obtaining data on BMI in childhood at the time of menarche as well as obesity-related comorbidities in adulthood.

### Limitations

The findings of the present study need to be seen in light of several limitations. First and foremost, this was a cross-sectional and not a population-based survey with a representative sample. The obtained results, therefore, should be extrapolated with caution. Other limitation factors also need to be stressed: self-reported age at menarche of the study participants, the lack of data on childhood obesity and the impossibility of explaining the effect of BMI in childhood and puberty on obesity in adult age. The association between BMI and obesity-related comorbidities was also excluded from the analysis.

## 5. Conclusions

Although the average age at menarche in young females in the region of Vojvodina falls within the category of normal menarcheal age and is comparable with internationally reported values, the study findings are worrying as more than a quarter of females had menarche at an early age and more than a fifth of them reported a late menarche onset. Overall, the obtained results point to a significant negative association between the age at menarche and general and abdominal obesity, which is congruent with the reports of other similar studies. However, obesity in adult women who experienced early menarche is associated with various other factors and the identification of these factors is very important from both individual and public health aspects. The results of this research highlight the need to monitor the health of women who experienced early menarche right from childhood or adolescence and to work on the education on healthy lifestyle and obesity prevention.

## Figures and Tables

**Table 1:** Demographic and anthropometric characteristics.

	Mean	SD	Minimum	Maximum
Age (years)	22.56	0.81	21.00	25.10
Height (cm)	166.91	6.86	155.00	184.50
Weight (kg)	63.57	9.45	46.70	89.50
WC (cm)	74.74	8.46	60.00	105.50
HC (cm)	99.61	11.18	75.60	128.70
BMI (kg/m^2^)	22.86	3.50	16.63	32.97
WHR	0.75	0.06	0.62	0.90
WHtR	0.45	0.06	0.34	0.64

SD: standard deviation; BMI: body mass index; WC: waist circumference; HC: hip circumference; WHR: waist-to-hip ratio; WHtR: waist-to-height ratio.

**Table 2:** Mean values of indicators of general and abdominal obesity and height in relation to early and late age at menarche.

	˂12 Years (*n* = 26) ^#^	≥12 Years (*n* = 76)	Difference	≤14 Years (*n* = 80)	>14 Years (*n* = 22) ^#^	Difference
Height	166.16 ± 6.05	167.17 ± 6.93	1.1 cm	166.28 ± 6.86	169.21 ± 5.65	2.92 cm
Height	166.38 ± 4.97	167.09 ± 7.41	0.7 cm	166.82 ± 7.06	167.24 ± 6.21	0.4 cm
BMI (kg/m^2^)	24.83 ± 4.64 ^a^	22.20 ± 2.73	2.63 kg/m^2^	23.26 ± 3.53	21.40 ± 3.01	1.86 kg/m^2^
WC (cm)	79.38 ± 11.80 ^a^	73.15 ± 6.21	6.23 cm	76.06 ± 8.41	69.95 ± 6.51	6.11 cm
WHR	0.76 ± 0.07	0.75 ± 0.05	0.01	0.76 ± 0.06	0.74 ± 0.06	0.02
WHtR	0.48 ± 0.08 ^b^	0.44 ± 0.04	0.04	0.46 ± 0.06	0.42 ± 0.05	0.04

^#^ Mann–Whitney U test of early and late age at menarche; ^a^: *p* ˂ 0.01; ^b^: *p* ˂ 0.05; BMI: body mass index; WC: waist circumference; WHR: waist-to-hip ratio; WHtR: waist-to-height ratio.

**Table 3:** Age at menarche in relation to general and abdominal obesity ((*n* (%)).

	Age at Menarche (*n* (%))			
	Early	Late	Mean (SD)	Median (SE)
BMI < 18.50 kg/m^2^ (*n* = 9)	1 (11.11)	5 (55.56)	13.84 (1.31)	14.10 (0.44)
BMI (18.50–24.99 kg/m^2^) (*n* = 70)	13 (18.57)	15 (21.43)	12.86 (1.58)	12.69 (0.19)
BMI (25–30 kg/m^2^) (*n* = 18)	7 (38.89)	2 (11.11)	12.45 (1.16)	12.16 (0.27)
BMI > 30 kg/m^2^ (*n* = 5)	5 (100)	-	11.39 (0.60)	11.79 (0.27)
WC ≤ 80 cm kg/m^2^ (*n* = 79)	13 (16.46)	20 (25.32)	13.00 (1.47)	12.75 (0.16)
WC > 80 cm (*n* = 23)	13 (56.52)	2 (8.70)	12.12 (1.53)	11.85 (0.32)
WHR ≤ 0.80 (*n* = 80)	17 (21.25)	18 (22.5)	12.89 (1.50)	12.64 (0.17)
WHR > 0.80 (*n* = 22)	9 (40.91)	4 (18.18)	12.48 (1.60)	12.60 (0.34)
WHtR < 0.40 (*n* = 14)	3 (21.43)	7 (50.00)	13.70 (1.78)	13.91 (0.48)
WHtR (0.40–0.49) (*n* = 69)	14 (20.29)	14 (20.29)	12.82 (1.42)	12.65 (0.17)
WHtR ≥ 0.50 (*n* = 19)	9 (47.37)	1 (5.26)	12.09 (1.38)	12.10 (0.32)
Total (N = 102)	26 (25.49)	22 (20.57)	12.80 (1.53)	12.62 (0.15)

SD: standard deviation; SE: standard error; BMI: body mass index; WC: waist circumference; WHR: waist-to-hip ratio; WHtR: waist-to-height ratio.

**Table 4:** The effect of age at menarche on height, BMI, WC, WHtR and WHR.

	R^2^	BETA	*p*-Value	95% CI	
				Lower	Upper
Height	0.151	0.151	0.131	−0.444	3.377
BMI	0.0.97	−0.312	*0.001*	−0.305	−0.075
WC	0.150	−0.387	*˂0.000*	−6.955	−2.490
WHR	0.021	−0.146	0.143	−0.029	0.004
WHtR	0.109	−0.331	*0.001*	−0.044	−0.012

R^2^ correlation coefficient; BETA: regression coefficients; *p*: significance level; *p*-value in italics: statistically significant values; 95% CI: 95% confidence interval; BMI: body mass index; WC: waist circumference; WHR: waist-to-hip ratio; WHtR: waist-to-height ratio.

**Table 5:** The effect of age at menarche on general and abdominal obesity.

	B	Wald	*p*-Value	OR	95% CI for OR	
					Lower	Upper
BMI	−0.355	4.483	*0.034*	0.701	0.505	0.974
WC	−0.418	5.700	*0.016*	0.658	0.469	0.925
WHR	−0.194	1.254	0.226	0.824	0.601	1.128
WHtR	−0.385	4.625	*0.032*	0.680	0.479	0.966

B: coefficient; OR: odds ratio; *p*: significance level; *p*-value in italics: statistically significant values; 95% CI: 95% confidence interval; BMI: body mass index; WC: waist circumference; WHR: waist-to-hip ratio; WHtR: waist-to-height ratio.

## Data Availability

Data are contained within the article and Supplementary Materials.

## Supplementary Materials

The following supporting information can be downloaded at: https://www.mdpi.com/article/10.3390/medicina60101711/s1, Questionnaire: The questionaire contains items on general and anthropometric traits of respondents.

## References

[1] Hillard P.J.A. (2008). Menstruation in adolescents: What’s normal?. Medscape J. Med..

[2] Arcoverde G.F., Prado L., Burgos M.G., Lima e Silva R., Andrade M.I. (2020). Early menarche and its association with anthropometric and body composition variables in young university students. Rev. Chil. Nutr..

[3] Kim H., Choe S.A., Lee S.J., Sung J. (2021). Causal relationship between the timing of menarche and young adult body mass index with consideration to a trend of consistently decreasing age at menarche. PLoS ONE.

[4] Tarannum F., Khalique N., Eram U. (2018). A community-based study on age of menarche among adolescent girls in Aligarh. Int. J. Community Med. Public Health.

[5] Żurawiecka M., Wronka I. (2021). Association between age at menarche and body mass index, waist circumference, waist to hip ratio, and waist to height ratio in adult women. Am. J. Hum. Biol..

[6] Charalampopoulos D., Mcloughlin A., Elks C.E., Ong K.K. (2014). Age at Menarche and Risks of All-Cause and Cardiovascular Death: A Systematic Review and Meta-Analysis. Am. J. Epidemiol..

[7] Ersoy B., Balkan C., Guay T., Onag A., Egemen A. (2004). Effects of different socioeconomic conditions on menarche in Turkish female students. Early Hum. Dev..

[8] Krieger N., Kiang M.V., Kosheleva A., Waterman P.D., Chen J.T., Beckfield J. (2015). Age at menarche: 50-year socioeconomic trends among US-born black and white women. Am. J. Public Health.

[9] de Keiser-Schrama S.M.P.F.M., Mul D. (2001). Trends in pubertal development in Europe. Hum. Reprod. Update.

[10] Rakić R., Puškaš V., Pavlica T. (2020). Menarche in adolescents from Vojvodina (the Republic of Serbia) in the period 2001–2019. Anthr. Anz..

[11] Mishra G.D., Cooper R., Kuh D. (2010). A life course approach to reproductive health: Theory and methods. Maturitas.

[12] Ota K., Yamagishi K., Kishida R., Kihara T., Cui R., Tamakoshi A., Hiroyasu I.H. (2023). Relationships between Age at Menarche and Risk of Cardiovascular Disease Mortality among Japanese Women: The Japan Collaborative Cohort Study for Evaluation of Cancer Risk (JACC) Study. J. Atheroscler. Thromb..

[13] Karlson E.W., Mandl L.A., Hankinson S.E., Grodstein F. (2004). Do breast-feeding and other reproductive factors influence future risk of rheumatoid arthritis? Results from the Nurses’ Health Study. Arthritis Rheum..

[14] Goldberg M., Aloisio A.A.D., O’Brien K.M., Zhao S., Sandler D.P. (2020). Pubertal timing and breast cancer risk in the Sister Study cohort. Breast Cancer Res..

[15] Key T.J., Verkasalo P.K., Banks E. (2001). Epidemiology of breast cancer. Lancet Oncol..

[16] Won J.C., Hong J.W., Noh J.H., Kim D.J. (2016). Association between age at menarche and risk factors for cardiovascular diseases in Korean women: The 2010 to 2013 Korea national health and nutrition examination survey. Medicine.

[17] Reinehr T., Roth C.L. (2019). Is there a causal relationship between obesity and puberty?. Lancet Child Adolesc. Health.

[18] Anyanwu O.U., Ibekwe R.C., Nwokocha A.R., Ibe C.B. (2016). An assessment of sexual maturation among school girls in Abakaliki Metropolis, Ebonyi State, South-East Nigeria. Niger. Postgrad. Med. J..

[19] Lian Q., Mao Y., Luo S., Zhang S., Tu X., Zuo X. (2019). Puberty timing associated with obesity and central obesity in Chinese Han girls. BMC Pediatr..

[20] Khadgawat R., Marwaha R.K., Mehan N., Surana V., Dabas A., Sreenivas V., Gaine M.A., Gupta N. (2016). Age of Onset of Puberty in Apparently Healthy School Girls from Northern India. Indian Pediatr..

[21] Huang A., Reinehr T., Roth C.L. (2020). Connections between obesity and puberty: Invited by Manuel tena-sempere, Cordoba. Curr. Opin. Endocr. Metab. Res..

[22] McTigue K.M., Garrett J.M., Popkin B.M. (2002). The natural history of the development of obesity in a cohort of young U.S. adults between 1981 and 1998. Ann. Intern. Med..

[23] Ahn J.H., Lim S.W., Song B.S., Seo J., Lee J.A., Kim D.H., Lim J.S. (2013). Age at menarche in the Korean female: Secular trends and relationship to adulthood body mass index. Ann. Pediatr. Endocrinol. Metab..

[24] Bubach S., Menezes A.M.B., Barros F.C., Wehrmeister F.C., Goncalves H., Assuncao M.C.F., Horta B.L. (2016). Impact of the age at menarche on body composition in adulthood: Result from two birth cohort studies. BMC Public Health.

[25] Gill D., Brewer C.F., Del Greco M.F., Sivakumaran P., Bowden J., Sheehan N.A., Minelli C. (2018). Age at menarche and adult body mass index: A Mendelian randomization study. Int. J. Obes..

[26] Dunger D.B., Ahmed M.L., Ong K.K. (2006). Early and late weight gain and the timing of puberty. Mol. Cell Endocrinol..

[27] Kaplowitz P.B. (2008). Link between body fat and the timing of puberty. Pediatrics.

[28] Prentice P., Viner R.M. (2013). Pubertal timing and adult obesity and cardiometabolic risk in women and men: A systematic review and meta-analysis. Int. J. Obes..

[29] Dreyfus J., Jacobs D.R., Mueller N., Schreiner P.J., Moran A., Carnethon M.R., Demerath E.W. (2015). Age at Menarche and Cardiometabolic Risk in Adulthood: The Coronary Artery Risk Development in Young Adults Study. J. Pediatr..

[30] Rafique N., AlSheikh M.H. (2019). Identifying menarcheal age and its association with body mass index in young Saudi females. Saud. Med. J..

[31] Das S., Dasgupta D. (2018). Association of body mass index and waist hip ratio with menstrual characteristics: A study among a group of adolescents of Kolkata city. Hum. Biolo Rev..

[32] WHO (2008). Waist Circumference and Waist–Hip Ratio: Report of a WHO Expert Consultation, Geneva.

[33] Ashwell M., Gibson S. (2009). Waist to height ratio is a simple and effective obesity screening tool for cardiovascular risk factors: Analysis of data from the British National Diet and nutrition survey of adults aged 19 to 64 years. Obes. Facts.

[34] Werneck A.O., Oyeyemi A.L.D.R. (2018). Association between age at menarche and blood pressure in adulthood: Is obesity an important mediator?. Hypertens. Res..

[35] Yang L., Li L., Millwood I.Y., Lewington S., Guo Y., Sherliker P., Peters S.A., Bian Z., Wu X., Yu M. (2017). Adiposity in relation to age at menarche and other reproductive factors among 300 000 Chinese women: Findings from China Kadoorie biobank study. Int. J. Epidemiol..

[36] Piras N., Bozzola M., Bianchin L., Bernasconi S., Bona G., Lorenzini G., Buzi F., Rigon F., Tonini G., De Sanctis V. (2020). The levelling-off of the secular trend of age at menarche among Italian. Heliyon.

[37] Ferrari V., Stefanucci S., Ciofi D., Stagi S. (2022). Analysis of the Timing of Puberty in a Recent Cohort of Italian Girls: Evidence for Earlier Onset Compared to Previous Studies. J. Pediatr. Adolesc. Gynecol..

[38] Joinson C., Heron J., Lewis G., Croudace T., Araya R. (2011). Timing of menarche and depressive symptoms in adolescent girls from a UK cohort. Br. J. Psychiatry.

[39] Al-Awadhi N., Al-Kandari N., Al-Hasan T., Al-Murjan D., Ali S., Al-Taiar A. (2013). Age at menarche and its relationship to body mass index among adolescent girls in Kuwait. BMC Public Health.

[40] Le-Ha C., Beilin L.J., Burrows S., Huang R.C., Hickey M., Mory T., Hart J.H. (2018). Age at menarche and childhood body mass index as predictors of cardio-metabolic risk in young adulthood: A prospective cohort study. PLoS ONE.

[41] Bralić I., Tahirović H., Matanić D., Vrdoljak O., Stojanović-Spehar S., Kovacić V., Blazeković-Milaković S. (2012). Association of early menarche age and overweight/obesity. J. Pediatr. Endocrinol. Metab..

[42] Lago M.J., Faerstein E., Sichieri R., Lopes C.S., Werneck G.L. (2007). Association between menarche age and final height in the Pró-Saúde study. Rev. Ass. Med. Bras..

[43] Pai L.F., Wang D.S., Hsu W.F., Huang S.W., Chung C.H., Chen S.J., Chien W.C., Chu D.M. (2022). New insights into precocious puberty and ADHD: A nationwide cohort study. Pediatr. Res..

[44] Kacerosky P. (2011). What Is the Importance of Age at Menarche on Adult Height Relative to Other Known Factors?. Bachelor’s Thesis.

[45] Sinclair D. (1989). Human Growth after Birth.

[46] Ritzén E.M., Nilsson O., Grigelioniene G., Holst M., Sävendahl L., Wroblewski J. (2000). Estrogens and human growth. J. Steroid Biochem. Mol. Biol..

[47] Juul A. (2001). The effects of oestrogens on linear bone growth. Hum. Reprod. Update.

[48] Sumi A., Iwase M., Nakamura U., Fujii H., Ohkuma T., Ide H., Jodai-Kitamura T., Komorita Y., Yoshinari M., Kitazono T. (2018). Impact of age at menarche on obesity and glycemic control in Japanese patients with type 2 diabetes: Fukuoka Diabetes Registry. J. Diabetes Investig..

[49] Pierce M.B., Leon D.A. (2005). Age at menarche and adult BMI in the Aberdeen children of the 1950s cohort study. Am. J. Clin. Nutr..

[50] Gopalakrishna P.K., Purushothaman S., Reghunath S., Pushkar B., King K.I. (2016). Association of age at menarche with body mass index and waist–hip ratio. Int. J. Med. Sci. Public Health.

[51] Asrullah M., L’Hoir M., Feskens E.J.M., Melse-Boonstra A. (2022). Trend in age at menarche and its association with body weight, body mass index and non-communicable disease prevalence in Indonesia: Evidence from the Indonesian Family Life Survey (IFLS). BMC Public Health.

[52] Freedman D.S., Khan L.K., Serdula M.K., Dietz W.H., Srinivasan S.R., Berenson G.S. (2003). The relation of menarcheal age to obesity in childhood and adulthood: The Bogalusa heart study. BMC Pediatr..

[53] Davison K.K., Susman E.J., Birch L.L. (2003). Percent body fat at age 5 predicts earlier pubertal development among girls at age 9. Pediatrics.

[54] Ahmed M.L., Ong K.K., Dunger D.B. (2009). Childhood obesity and the timing of puberty. Trends Endocrinol. Metab..

[55] Perry J.R., Day F., Elks C.E., Sulem P., Thompson D.J., Ferreira T., He C., Chasman D.I., Esko T., Thorleifsson G. (2014). Parent-of-origin-specific allelic associations among 106 genomic loci for age at menarche. Nature.

[56] da Costa R.R., Moreira M.T.M., Florêncio R.S., de Paula Pessoa V.L.M., Cestari V.R.F., Montesuma F.G. (2018). Overweight and Associated Factors in Young Adult Student Girls. Rev. Bras. Enferm..

[57] Cho H., Han J.W. (2023). Obesity-Related Factors in Adult Women with Early Menarche. Healthcare.

[58] Jokela M., Elovanio M., Kivimaki M. (2008). Lower fertility associated with obesity and underweight: The US National Longitudinal Survey of youth. Am. J. Clin. Nutr..

[59] Klenk J., Nagel G., Ulmer H., Strasak A., Concin H., Diem G., Rapp K. (2009). Body mass index and mortality: Results of a cohort of 184,697 adults in Austria. Eur. J. Epidemiol..

[60] Feng Y., Hong X., Wilker E., Li Z., Zhang W., Jin D. (2008). Effects of age at menarche, reproductive years, and menopause on metabolic risk factors for cardiovascular diseases. Atherosclerosis.

[61] Lakshman R., Forouhi N.G., Sharp S.J., Luben R., Bingham S.A., Khaw K.T., Wareham N.J., Ong K.K. (2009). Early age at menarche associated with cardiovascular disease and mortality. J. Clin. Endocrinol. Metab..

[62] Peters S.A.E., Huxley R.R., Woodward M. (2016). Women’s reproductive health factors and body adiposity: Findings from the UK Biobank. Int. J. Obes..

[63] Ibanez L., Ong K., De Zegher F., Marcos M.V., Del Rio L., Dunger D.B. (2003). Fat distribution in non-obese girls with and without precocious pubarche: Central adiposity related to insulinaemia and androgenaemia from prepuberty to postmenarche. Clin. Endocrinol..

[64] Mueller N.T., Pereira M.A., Demerath E.W., Dreyfus J.G., Maclehose R.F., Carr J.J., Terry J.G., Jacobs D.R.J. (2015). Earlier Menarche is Associated with Fatty Liver and Abdominal Ectopic Fat in Midlife, Independent of Young Adult BMI: The CARDIA Study. Obesity.

[65] Trikudanathan S., Pedley A., Massaro J.M., Hoffmann U., Seely E.W., Murabito J.M., Fox C.J. (2013). Association of female reproductive factors with body composition: The Framingham heart study. J. Clin. Endocrinol. Metab..

