# Supplementary material for: Association of Age at Menarche with General and Abdominal Obesity in Young Women
Source: Medicina (Kaunas). 2024 Oct 18;60(10):1711. doi: 10.3390/medicina60101711 (PMC11509626; doi:10.3390/medicina60101711)
Supplement: Supplementary file 1 [file medicina-60-01711-s001.zip › medicina-3225307-supplementary.pdf]

UPITNIK

Poštovane studentkinje,

Molimo vas da popunite anketni upitnik koji je pred vama i učestvujete u antropometrijskom merenju. Upitnik je anoniman, pa vas molimo da iskreno odgovorite. Podaci bi se koristili isključivo u naučne svrhe. Odluka za pristup ispitivanju je potpunoVaša, svojevoljna.

HVALA NA SARADNJI!

Ime i prezime ______________________________

Datum ispitivanja ___________________________ godine

Datum rođenja _____________________________ godine

Datum dobijanja menarhe ____________________ godine

Visina tela _________________________________cm

Masa tela __________________________________kg

Obim struka ________________________________ cm

Obim kukova _______________________________ cm

QUESTIONNAIRE

Dear students,

We kindly ask you to fill out this questionnaire and thus take part in an anthropometric survey. The questionnaire is anonymous, so please provide honest answers. The data collected will be used exclusively for scientific purposes. Your participation in the survey is voluntary.

THANK YOU FOR YOUR COOPERATION!

Name and surname ______________________________

Date of survey ___________________________

Date of birth _________________________________

Date of first menarche _____________________________

Body height __________________________________cm

Body weight __________________________________kg

Waist circumference ___________________________ cm

Hip circumference ____________________________ cm
